# Influence of substrate orientation on tadpoles' feeding efficiency

**DOI:** 10.1242/bio.037598

**Published:** 2018-12-21

**Authors:** Fabiane Santana Annibale, Verônica Thiemi Tsutae de Sousa, Carlos Eduardo de Sousa, Matthew D. Venesky, Denise de Cerqueira Rossa-Feres, Fausto Nomura, Richard J. Wassersug

**Affiliations:** 1 Departamento de Ecologia, Universidade Federal de Goiás, Goiânia, GO, 74690-900, Brazil; 2 Departamento de Zoologia e Botânica, Universidade Estadual Paulista “Júlio de Mesquita Filho”, São José do Rio Preto, SP, 15054-000, Brazil; 3 Department of Biology, Allegheny College, Meadville, PA, 16335, USA; 4 Department of Cellular & Physiological Sciences, University of British Columbia, Vancouver, BC, V6T 1Z3, Canada

**Keywords:** Ecomorphology, Substrate angle, Anuran larvae, Feeding behavior, Niche partitioning, Oral morphology

## Abstract

In nature, tadpoles encounter food on substrates oriented at different angles (e.g. vertically along stems, horizontally on the bottom of the pond). We manipulated the orientation of food-covered surfaces to test how different orientations of surfaces affect tadpoles' feeding efficiency. We studied taxa that differed in the oral morphology of their larvae and position in the water column. We hypothesized that species would differ in their ability to graze upon surfaces at different orientations and that differences in the tadpoles' feeding ability would result in different growth rates. The orientation of food-covered surfaces did not affect the growth rate of bottom-dwelling tadpoles (whose growth rate varied only between species). Among midwater tadpoles, some species appear to have a generalist strategy and experienced a high relative growth rate on numerous substrate orientations, whereas others achieved high growth rates only on flat substrates (i.e. at 0° and 180°). We conclude that oral morphology constrains tadpoles' ability to feed at different substrate orientations, and this could lead to niche partitioning in structurally complex aquatic environments. Because physical parameters of the environment can affect tadpoles' growth rate, characterizing these features might help us better understand how competition structures tadpole assemblages.

## Abstract (precis)

**Summary:** We provide new insights on species' niche partitioning as we show that the orientation of substrates can affect the growth rates of tadpoles with different oral morphologies.

## INTRODUCTION

The external oral apparatus of most anuran larvae is comprised of a soft, marginally papillated oral disc that surrounds keratinized jaw sheaths and rows of keratinized labial teeth (McDiarmid and Altig, 1999). The function of the marginal papillae is not well established, but some studies suggest that they can facilitate certain tadpoles' ability to adhere to substrates in lotic environments (e.g. Altig and Johnston, 1989). However, the keratinized structures of tadpoles are well described because of their use in anuran systematics (e.g. Orton, 1953; Starrett, 1973; Vera Candioti, 2007). The keratinized jaw sheaths and labial tooth rows lie anterior and posterior to the oral opening. Those keratinized structures are used by tadpoles to scrape or bite organic material off the substrate as food (Wassersug and Yamashita, 2001). The keratinized and soft structures vary substantially in complexity among species as both labial tooth rows and marginal papillae can vary in size, arrangement, and configuration (McDiarmid and Altig, 1999; Altig, 2007).

Morphological variation in oral structures of vertebrates usually reflect the resources that are consumed [e.g. bird beaks reflect the type of food they eat (Darwin, 1859; MacArthur, 1958)]. Consequently, morphological variation in oral structures typically correlates with dietary niche (Begon et al., 2006). However, this is not the case for many tadpoles. Tadpoles of different species, each with diverse oral structures, can coexist in a single pond during the same season, yet individuals of each species have similar gut contents (Rossa-Feres et al., 2004; Prado et al., 2009). This finding suggests that tadpoles are dietary generalists, and it raises questions related to resource partitioning in diverse community assemblages of tadpoles. Are tadpoles of many species able to use the same resources in the same place and at the same time without one species outcompeting the other?

Our working hypothesis is that anuran larvae divide up the environment not necessarily in terms of the food that they feed on, but in their efficiency for grazing upon various surfaces, each of which have different intrinsic physical properties. These substrate properties could include orientation, firmness and texture. Here we explore the first of these properties and ask, ‘how does the orientation of substrates affect tadpoles’ feeding efficiency?’ When anuran larvae differ in their efficiency to feed on a particular surface, those efficiency differences might affect growth and development. If so, less efficient species may explore other microhabitats to avoid competition with more efficient species (Alford, 1986).

Morphological characteristics related to locomotion and feeding are key to a species’ ability to exploit the physical dimensions of a microhabitat (Higham, 2007). For example, the morphology of lizard digits is associated with climbing ability and thereby determines where they are able to feed [e.g. on the side of rocks or the underside of branches while upside down (Irschick et al., 1996; Higham and Jayne, 2004)]. This relationship between locomotor morphology and substrate utilization can apply to aquatic vertebrates as well. Aquatic salamanders are able to adjust the elevation of their heads to capture prey in the water in different orientations (Shaffer and Lauder, 1985). Similarly, cichlid fish have the ability to swim in different positions and to adjust the orientation of their bodies and oral apparatus to acquire food from substrates oriented at different angles (Rupp and Hulsey, 2014). Among tadpoles, differences in feeding efficiency exist even for species that have similar feeding behavior. When feeding upon suspended particles, species differ in rates of particle capture and also in efficiency at gathering particles of different sizes (Seale and Wassersug, 1979; Seale et al., 1982). Thus, it is possible that variation in the keratinized oral structures of tadpoles either limits or facilitates their ability to remove food from substrates at different orientations. If so, differences in feeding efficiencies on various substrates may thus both force and enable tadpoles to partition the environment even when the food matter growing on the surfaces may be abundant and the same.

We manipulated the angle at which food was offered to tadpoles and tested how this angle (i.e. orientation) affected the feeding efficiency of tadpoles from several species that differ in their oral morphology. We hypothesized that species (1) differ in their ability to graze in surfaces at different orientations and (2) partition their habitat use based on the orientation of the surfaces on which they graze most efficiently. We predicted that bottom-dwelling species would be more efficient removing food from horizontal surfaces, as these tadpoles are usually negatively buoyant and typically forage on the bottom of ponds (Altig and Johnston, 1989; McDiarmid and Altig, 1999). Conversely, we expected that tadpoles that are more commonly found in the water column would be better able to acquire food from vertical and sloping surfaces, as they usually graze upon stems and leaves above the bottom surface. We also predicted that tadpoles that share the same microhabitat, but have different oral morphologies, would differ in their feeding efficiency in relation to substrate orientation. Species, for example, with smaller oral discs and fewer keratinized structures may have more flexible oral discs (Altig, 2006). These more flexible disks may permit them to feed more efficiently upon more contoured surfaces, such as the vertical stems of aquatic plants. Conversely, species with a higher number of keratinized structures usually have a larger oral apparatus, which is in general ventrally oriented, so they may be more efficient feeding on horizontally oriented substrates. Finally, because species that occur at the same depth of the water column and have similar oral morphologies usually share the same resources, we predicted that these species would feed on the same orientations with a similar efficiency.

## RESULTS

Food consumption rate was a positive predictor of tadpole mass (all prediction tests: *P*<0.05, Table 1). The substrate orientation influenced the feeding efficiency of tadpoles that have similar external oral morphology, but differ in where they are found within a pond. For example, tadpoles of *Leptodactylus fuscus* and *Scinax fuscovarius* (benthic and nektonic, respectively) have similar oral morphology, but their relative mass gain depended on the substrate orientation. However, substrate orientation did not affect the relative mass gain of species that occur within the same microhabitat (e.g. bottom-dwelling tadpoles). Among tadpoles that differ in their oral morphology, but occur in the same microhabitat, we found a species by treatment interaction. This indicates that some species had similar growth rates regardless the orientation of substrates, but others were more efficient when feeding in specific orientations.

**Table 1.**
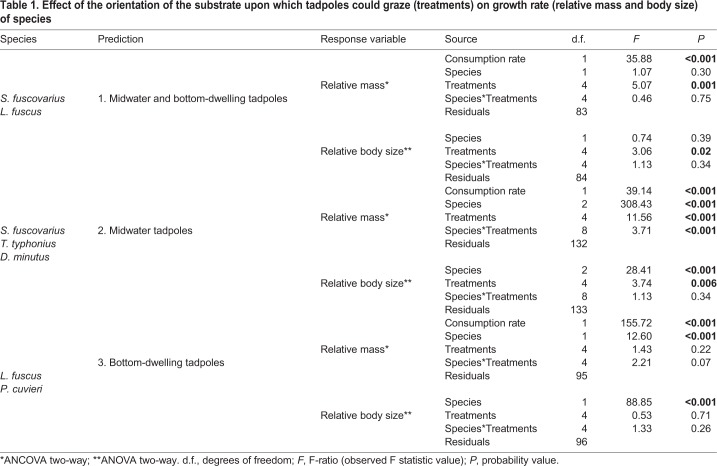
**Effect of the orientation of the substrate upon which tadpoles could graze (treatments) on growth rate (relative mass and body size) of species**

### Bottom-dwelling versus midwater tadpoles with similar oral morphology

**Fig. 1.**
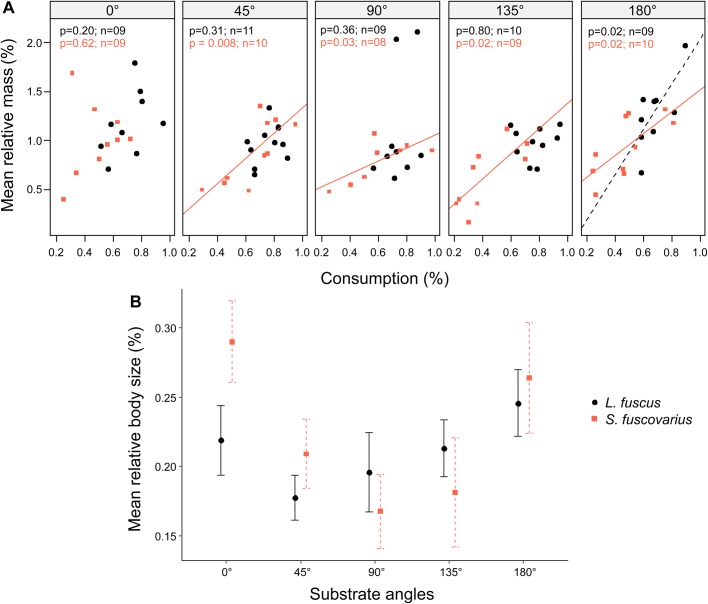
**Effect of substrate orientation on growth rate of bottom-dwelling (*L. fuscus*) and midwater (*S. fuscovarius*) tadpoles that have similar external oral morphology.** The legend identifies the species for both graphs. (A) Relative mass versus percentage of food consumed by orientation of the substrates (two-way ANCOVA). Solid and dashed lines represent significant regression lines between relative mass and food consumption by treatment. The probability values (*P*) for this relationship and the number of tadpoles tested (*n*) are presented in respective colors to each species in the panels. Symbols (circles and squares) represent each individual. (B) Relative body size versus percentage of food consumed by orientation of the substrates (two-way ANOVA). Symbols represent mean values and bars indicate standard error. Species had similar growth rates (A,B, *P*>0.05), but performed better feeding on horizontal substrates (A,B, *P*<0.05).

When comparing *L. fuscus* and *S. fuscovarius*, substrate orientation significantly affected the tadpoles' relative mass (*F*=5.07, *P*=0.001). Neither species nor the interaction between species and treatment affected relative mass change (Table 1). Tadpoles of both species experienced the greatest change in mass when feeding on horizontal angles (i.e. 0° and 180°; Fig. 1A). Specifically, tadpoles gained 30% more mass when feeding on substrates at 0° compared to tadpoles that fed on substrates at 135° (Fischer test, *P*=0.002).

We found differences in tadpoles’ relative body length among substrate orientations (*F*=3.05, *P*=0.02, Fig. 1B, Table 1), but not between species (Table 1). Tadpoles feeding upon both substrates at 0° and 180° increased 7% more than tadpoles grazing on substrates at 45° (Fischer test, *P*=0.02), 90° (Fischer test, *P*=0.01) and 135° (Fischer test, *P*=0.04).

### Similar microhabitat (midwater) but different oral morphology

**Fig. 2.**
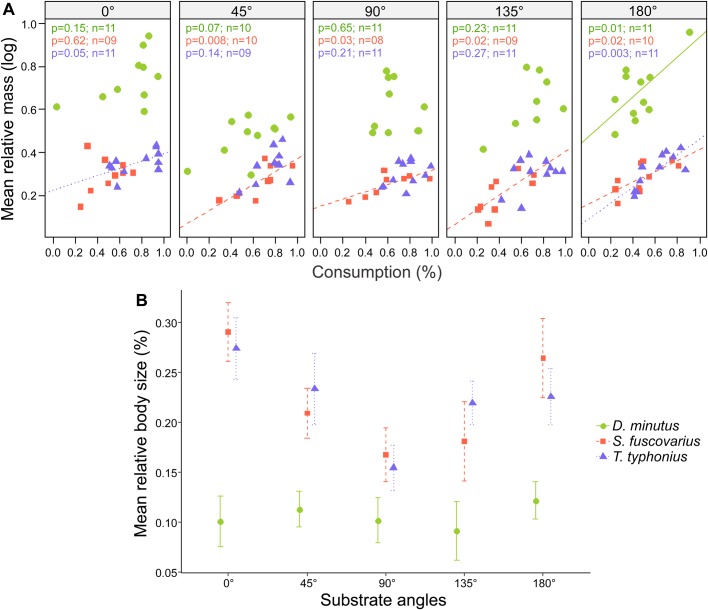
**Effect of substrate orientation on growth rate of midwater tadpoles with different oral configurations.** The legend for species applies to both graphs. (A) Relative mass versus percentage of food consumed by substrate angles (two-way ANCOVA). Solid and dashed lines represent significant regression lines between relative mass and food consumption by treatment. The probability values (*P*) of this relationship and the number of tadpoles tested (*n*) are presented in respective colors to each species in the panels. Symbols (circles, triangles and squares) represent each individual. (B) Relative size versus percentage of food consumed by orientation of the surfaces (two-way ANOVA). Symbols represent mean values and bars indicate standard error. The orientation of the substrates affects not only the ability of tadpoles to feed, but also their growth rates. Note that some species were specialists and had higher growth rates depending on the substrate orientation. Tadpoles of *D. minutus* were generalists and presented a higher increase in relative mass despite the angle of substrate (A, interaction effect between species and substrate orientations, two-way ANCOVA, *P*<0.001). However, this species had the least increase in body size (but a high increase in tail length). See Results for more details.

The interaction between substrate angles and species significantly accounted for the variation in tadpoles' mass (*F*=3.71, *P*<0.001, Fig. 2A, Table 1). Tadpoles of *Trachycephalus typhonius* and *S. fuscovarius* did not differ in their relative mass feeding on substrates at all the angles tested. Tadpoles of *Dendropsophus minutus* had the poorest performance when grazing on substrates at 45° (i.e. half the mass of tadpoles feeding at horizontal angles, Table S1). These tadpoles, which have the simplest oral disc, gained more mass than the other two species.

When we analyzed the relative lengths of tadpoles, we found variation among species (*F*=28.41, *P*<0.001, Fig. 2B, Table 1) and among treatments (*F*=3.74, *P*=0.006), but not their interaction. Among species, tadpoles of *D. minutus* had the least increase in size compared to *T. typhonius* (Fischer test, *P*<0.001) and *S. fuscovarius* tadpoles (Fischer test, *P*<0.001). We did not find differences in relative growth between *T. typhonius* and *S. fuscovarius* (Fischer test, *P*=0.84). Among orientations, tadpoles feeding at 0° increased 8% more than those at 90° (Fischer test, *P*<0.001) and 5% more than those at 135° (Fischer test, *P*=0.03). Also, tadpoles feeding at 180° increased 6% more than tadpoles feeding at 90° (Fischer test, *P*=0.006).

Due to this intriguing result for *D. minutus* (i.e. the highest mass gain and the lowest body growth among species), we investigated whether the low increase in body size was compensated for by an increase in tail lengths (using Eqn1). We only used tail measurements from individuals whose tail was not folded behind their body in the pictures; (*n*=43). Indeed, we verified that their tails increased around 30% in length and this was 20% greater than the increase in body size.

### Similar microhabitat (bottom) and oral morphology

**Fig. 3.**
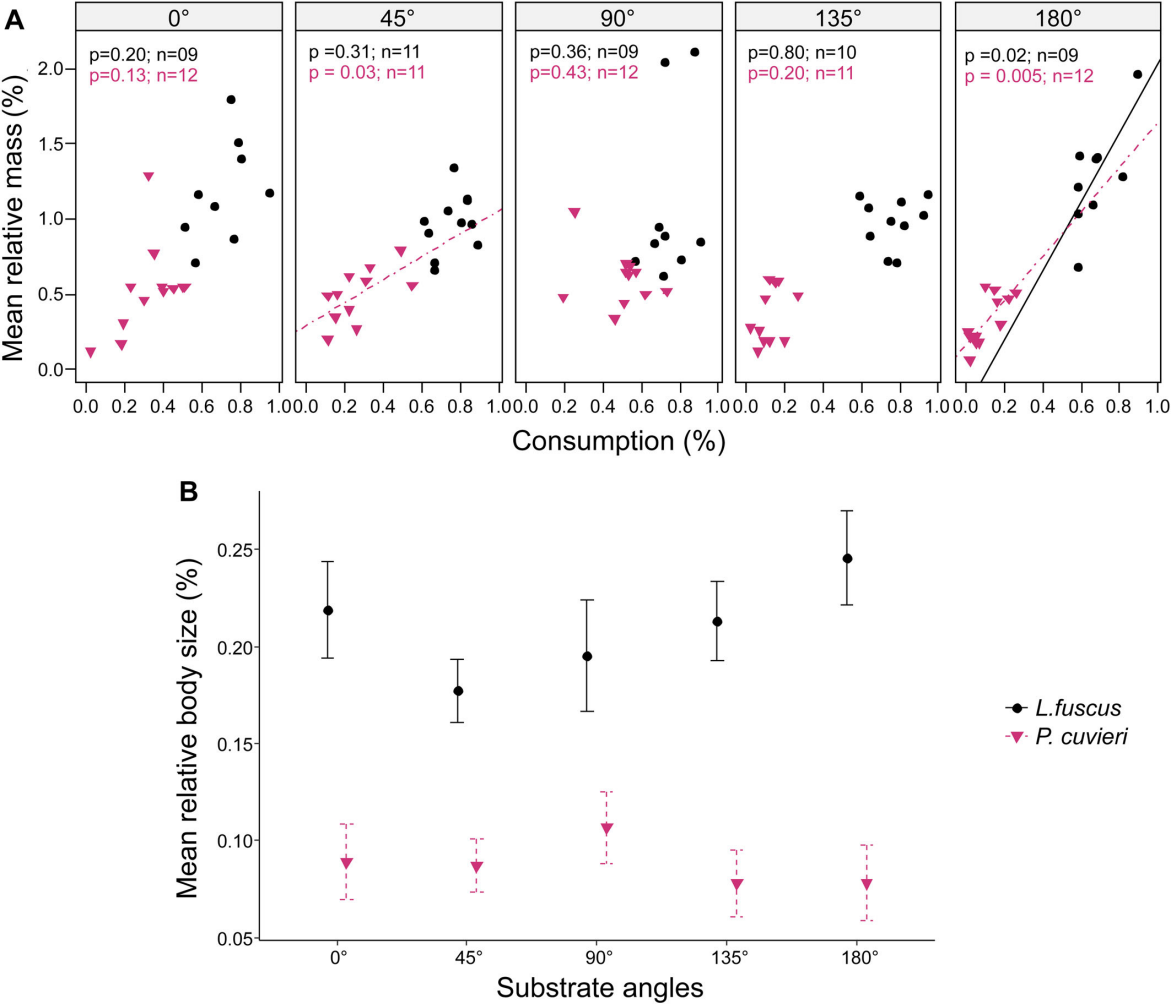
**Effect of substrate orientation on growth rate for bottom-dwelling tadpoles with similar oral configuration.** The legend for species applies to both graphs. (A) Relative mass versus percentage of food consumed by treatment. Solid and dashed lines represent significant regression lines. Symbols (circles and inverted triangles) represent each individual. Probability values (*P*) of the relationship between relative mass and consumption and the number of tadpoles tested (*n*) are presented in respective colors to each species. (B) Relative size versus percentage of food consumed by substrate angles (two-way ANOVA). Symbols represent mean values and bars indicate standard error. Species were equally capable of exploring substrates at the same orientations (A,B, *P*>0.05), with differences in growth rate only between species (A,B, *P*<0.001).

The relative mass gained by bottom-dwelling tadpoles with similar oral morphology showed no effect of treatments (*F*=1.43, *P*=0.22, Fig. 3A, Table 1). Between species, tadpoles of *L. fuscus* doubled their mass, while the mass gain for tadpoles of *Physalaemus cuvieri* was only half that of *L. fuscus* tadpoles (*F*=12.60, *P*<0.001, Table S1).

Also, treatments had no effect on tadpoles’ relative body size (*F*=0.53, *P*=0.71), but tadpoles of *L. fuscus* increased 10% more than those of *P. cuvieri* (*F*=88.84, *P*<00.1, Fig. 3B, Table 1).

### Other behavioral observations

Tadpoles moved their tails much less frequently when feeding at the angles of 0° and 180°, floating while grazing. In contrast, at 45°, 90° and 135° tadpoles swam up and down while grazing.

## DISCUSSION

Theory suggests that morphologically similar species who use the same resources have the highest competitive potential (MacArthur and Levins, 1967) and may fail to coexist (Gause, 1934). Among tadpoles, competition for nutritional resources has controversially been considered weak (e.g. Heyer, 1976) or of secondary importance in natural settings (Skelly and Kiesecker, 2001; Schiesari et al., 2009). That is because of a general perception that tadpoles are not particularly selective in terms of when and where they acquire food, feeding in a rather continuously and non-discriminatory fashion on the most available resource (Lajmanovich, 2000). This perception is endorsed by the fact that, despite diversity in oral morphology, much of what is found in tadpole alimentary tracts are the same common and abundant food items (Rossa-Feres et al., 2004). This suggests that food is not below a limiting threshold to force competition (Heyer, 1976).

There is evidence, however, that tadpoles can select and compete for resources based on the nutritional properties of the food and/or by the characteristics of the substrates where they graze (e.g. Kupferberg, 1997; Pfennig et al., 2007; Ramamonjisoa et al., 2017). In our study we provide new insights on niche partitioning among tadpoles as we show that the orientation of substrates can affect tadpoles’ growth rates. Regardless of whether food type is a limiting resource for tadpoles or not, the physical properties of the substrates where they forage can be. Although we used an experimental approach to control for the substrate orientation, food can be found on the surfaces of macrophytes, rocks or even suspended in the water column and thus oriented in all sorts of directions. Our data indicate that tadpoles may thus partition the habitat in terms of the surface orientations upon which they most efficiently graze.

Overall, all tadpoles were able to feed and grow on substrates at all orientations, however some species (e.g. *T. Typhonius* and *S. fuscovarius*) showed the best feeding efficiencies when foraging on horizontal substrates (i.e. at the angles of 0° and 180°). Although we did not measure any functional morphological traits, it is plausible that tadpoles of these species can make kinematic adjustments of their oral apparatus at these substrate angles to provide the best contact with the substrate (McDiarmid and Altig, 1999). Furthermore tadpoles appear to be able to feed at these angles with little active swimming, because they exhibited little tail movement while grazing. The size of their lungs and the adjustment of their center of buoyancy may provide more stability and reduce energy costs (Alexander, 1966), influencing tadpoles' efforts to get the optimal position for grazing. In contrast, tadpoles had lower growth rates when feeding on substrates at 45°, 90° and 135°. In these treatments, tadpoles swam more while grazing. The lower growth rates may thus reflect an increased energetic cost for tadpoles feeding at these angles and not kinematic adjustments.

### Bottom-dwelling versus midwater tadpoles with similar oral morphology

Body shape commonly reflects tadpoles' preferred microhabitat (Marques and Nomura, 2015; Queiroz et al., 2015), but this may not correlate closely with the substrate orientations upon which tadpoles feed most efficiently. Tadpoles of *S. fuscovarius* are usually found close to leaves and aquatic plants in midwater (Schulze et al., 2015). Yet, contrary to our prediction, they exhibited the least efficiency on substrates positioned at a 90° angle. In fact, bottom-dwelling and midwater tadpoles had similar growth rates when feeding on horizontal substrates (similar relative mass at 0° and relative body size at 0° and 180°). In contrast, tadpoles of *L. fuscus* have higher feeding efficiencies grazing at all the substrate orientations – tadpoles’ mass doubled at all the angles tested. This reflects a large niche breadth in terms of orientation of substrates.

These species differ in overall external morphology, but have similar oral morphology. Besides, their oral discs are in the anteroventral position (Rossa-Feres and Nomura, 2006). This suggests that the tadpoles' overall morphology is of secondary importance in influencing their ability to feed at different orientations. Differences in external body morphologies may be important in helping the larvae to orient to surfaces upon which they graze – specifically maintaining an optimal body position while grazing. Body shape may also reflect adaptions to factors other than feeding behavior. For example, midwater tadpoles usually have deep tails with high fins and a flagellum at the tip. This design may aid maneuverability for the tadpole when under predator attack (Wassersug, 1989).

### Similar microhabitat (midwater) but different oral morphology

We found statistically significant differences in feeding efficiency among tadpoles that live in the same microhabitat but have different oral morphology when allowed to graze on substrates at different orientations. This suggests that interspecific variation in mouthparts influences the ability of tadpoles of different species to forage, depending on characteristics of the physical habitat. This in turn, reflects niche partitioning among species.

Tadpoles of *T. typhonius* and *S. fuscovarius* were equally able to gain mass feeding at all orientations of substrates, but they had higher body lengths in specific angles. In particular, the increase in body size of both species was higher for tadpoles feeding on horizontal surfaces and for tadpoles of *T. typhonius* on substrates at 45°. This indicates that these tadpoles can be more restricted in their microhabitats, feeding preferentially on surfaces where they benefit both by gaining mass and growing more. Tadpoles of *D. minutus*, which have the simplest oral morphology, were generalists in terms of their effectiveness in grazing on substrates at different orientations, with similar growth rates at all angles of substrates (despite reduced relative mass gain at 45°). These tadpoles achieved higher relative mass than the other species, but, inversely, had smaller body lengths. This was compensated for by greater investment in tail growth, which was greater than body growth. Thus, tadpoles of *D. minutus* differed from the other midwater species in their strategy to invest in growth of different parts of their body.

Although these species co-occur in close proximity to each other in ponds (Prado et al., 2009), they can forage for the same nutritional resources in functionally different ways (e.g. Rupp and Hulsey, 2014). As such, our results challenge the presumption that competition is low for tadpoles (Heyer, 1976). Even if tadpoles do not partition the environment in terms of what they ingest, our data demonstrate that they may differ in terms of where they can most effectively acquire food and grow well. When tadpoles explore the same alimentary resources, variation in performance is the most likely factor to producing shifts in the microhabitats used by tadpoles for foraging and consequently their feeding niche (e.g. Pfennig and Murphy, 2002; Pfennig et al., 2007). Contrary to Heyer (1976) then, we suggest that competition may be a factor driving the evolution of diversity in tadpole mouthparts and thus lead to species segregation among anuran larvae.

### Similar microhabitat (bottom) and oral morphology

As the bottom-dwelling species that we examined are morphologically similar in body shape and oral morphology, we predicted that tadpoles would be equally efficient in feeding on the variously oriented substrates. This was corroborated. Both species (*P. cuvieri* and *L. fuscus*) were grazing generalists in terms of the orientations of substrates. This ability to graze and grow successfully upon substrates despite their orientation might represent an additional and important factor for these tadpoles to survive in a variety of habitats where they are found. These habitats include marginal areas of deep water ponds, shallow ponds (Queiroz et al., 2015; Schulze et al., 2015) and even in puddles close to streams (e.g. *P. cuvieri* in 1 cm deep puddles, Eterovick and Sazima, 2000; and in the very shallow margins of rivulets, D.C.R.-F., unpublished).

We did observe some differences in feeding efficiency between the tadpoles of *L. fuscus* and those of *P. cuvieri*. Tadpoles of *L. fuscus* consumed more food and also had higher growth rates than the *P. cuvieri* larvae. Possibly other factors contribute to differences in feeding efficiency between these species, such as behavior – e.g. levels of activity can have a straightforward relationship with consumption of food (Anholt and Werner, 1995). Other variables might also be important for these species in defining their preferred feeding niche (e.g. vegetation, Waringer-Löschenkohl, 1988; pH, Devi et al., 2016; ontogeny, Glos et al., 2017).

### Conclusions and future directions

In our study, tadpoles were able to feed on substrates at all angles tested, however, with different efficiency. Differences in feeding efficiency and morphological specializations can play an important role in nutritional acquisition in structurally complex environments (Higham, 2007). Morphological variation in the tadpole oral apparatus can be key to their feeding efficiency. The differences that we found in the tadpoles' feeding performance when foraging on substrates at different angles may thus be an important aspect of niche partitioning for the species.

In summary, this study advances our understanding of the ecology of neotropical tadpoles, which are poorly studied despite their high taxonomic diversity (especially in Brazil; Rossa-Feres et al., 2015). Our experiments demonstrate that the orientation of substrates influences tadpoles’ feeding efficiency, which is fundamental for tadpole survival. Whereas some species (e.g. bottom-dwelling tadpoles and larvae of *D. minutus*) are generalists in terms of the orientation of the substrate upon which they feed efficiently, other species are specialists and perform better feeding on substrates at specific orientations. This indicates that species are not only segregated by preferred position in the water column (e.g. bottom/midwater/surface), but also by the orientation of substrates upon which they graze.

We suggest that competition has been underappreciated in studies with anuran larvae. Even if food type is not a limiting resource for tadpoles, their efficiency to capture food depends on physical properties of substrates, as we have demonstrated with orientations of surfaces in our experiments. Competition can be reduced for tadpoles by using different portions of the same substrate (e.g. the stems versus the underside of a leaf) and at the same depth of the water column. Thus, tadpoles may partition aquatic habitats more than previously presumed – e.g. not only in terms of their depths in the water column, but also due to the orientations of substrates upon which they feed.

In our study we were not able to elucidate intrinsic differences in nutrient assimilation among the species. Together with how much food the larvae are able to remove from the substrates and ingest, nutrient assimilation may influence their growth rates. Such basic data about tadpoles' biology would fill major gaps in our knowledge of anuran larval ecology and evolution. Similarly, kinematic data on how tadpoles position their oral disc, body and tail while grazing on different substrates would further advance our understanding of what behavioral factors influence tadpole feeding efficiency. Future studies should test more species and explore other physical properties of the microhabitat that may be relevant to tadpole resource partitioning. These could include, for example: time of day, surface stiffness and surface roughness. Such research would greatly advance our understanding of how tadpoles of different species coexist.

## MATERIALS AND METHODS

### Species

We tested our hypotheses using tadpoles of five anuran species: *Physalaemus cuvieri* Fitzinger, 1826, *Leptodactylus fuscus* (Schneider, 1799), *Scinax fuscovarius* (Lutz, 1925), *Dendropsophus minutus* (Peters, 1872) and *Trachycephalus typhonius* (Linnaeus, 1758). These species are usually classified in two guilds – benthic (*P. cuvieri* and *L. fuscus*) and nektonic (*S. fuscovarius*, *D. minutus* and *T. typhonius*) (Rossa-Feres and Nomura, 2006; Marques and Nomura, 2015). This classification is based primarily on the position where tadpoles are found in the water body (benthic, bottom; nektonic, midwater) and on their external morphology. Benthic tadpoles typically have dorsoventrally compressed bodies, shallow fins and dorsal eyes. Nektonic tadpoles have more laterally compressed bodies, deep fins, the presence of a flagellum at the tip of the tail and lateral eyes (McDiarmid and Altig, 1999).

**Fig. 4.**
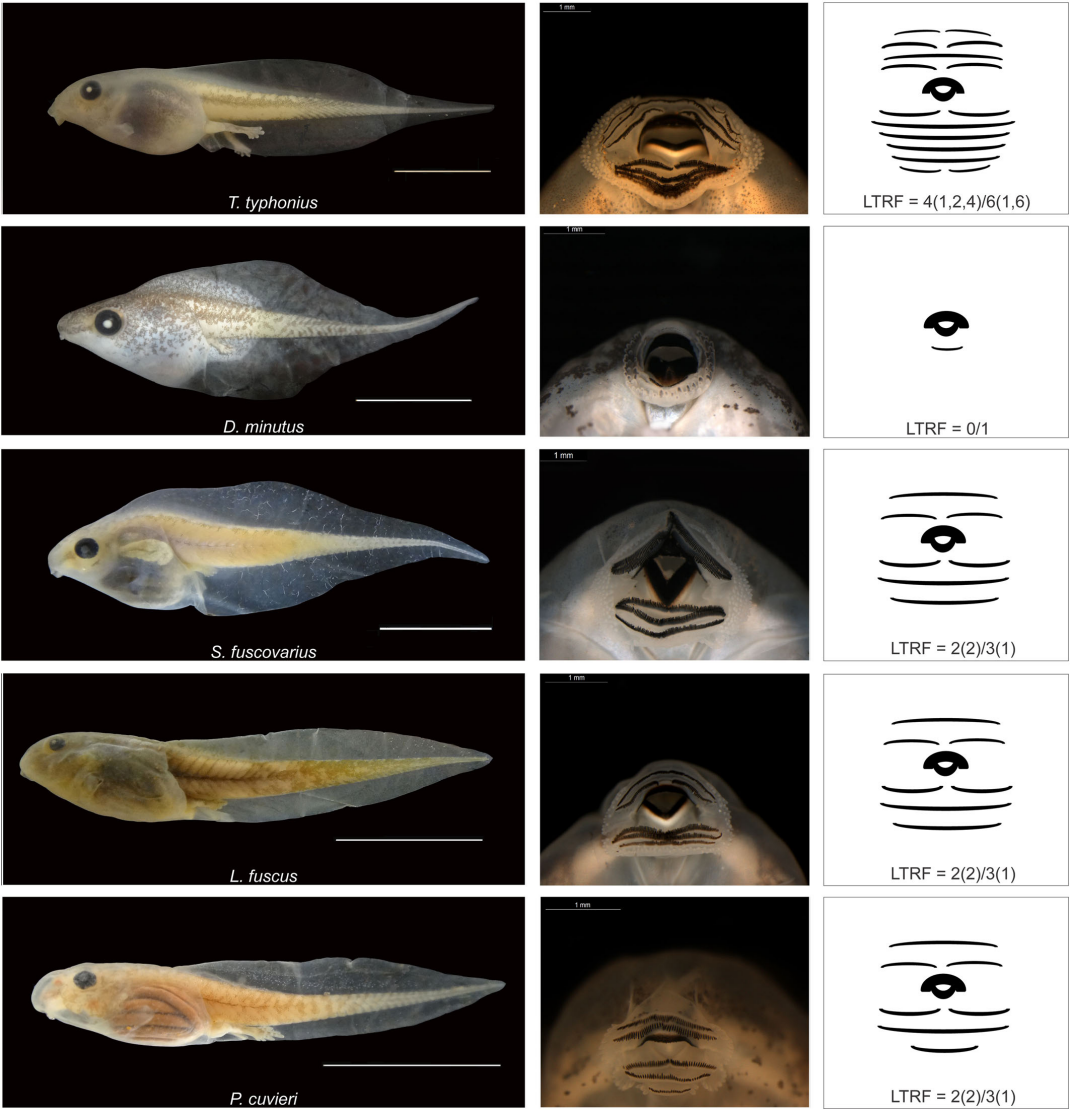
**The species tested in the study (left), the oral apparatus of tadpoles (center) and a schematic illustration (right) of the tadpoles' keratinized structures base the labial tooth row formula (LTRF).** Besides differences in LTRF (indicated in the figure), midwater tadpoles also differ in marginal papillae row configuration: *T. typhonius* with two marginal papillae rows; *D. minutus* with one ventral and two lateral marginal papillae rows; and *S. fuscovarius* with one marginal papillae row. Also, the oral disc of *D. minutus* tadpoles is terminally positioned. The oral morphology of bottom-dwelling tadpoles (*L. fuscus* and *P. cuvieri*) is similar to *S. fuscovarius*. However tadpoles of *P. cuvieri* have a more ventrally positioned oral disc and their third posterior tooth row is one third smaller than the other posterior tooth rows (in Rossa-Feres and Nomura, 2006). Photographs are on the same scale and were provided by K.O.R. Picheli.

We selected these species based on the morphology of their external oral structures (Fig. 4). We used their labial tooth row formula (LTRF) to characterize variation in keratinized structures among species because the number and the arrangement of the rows in the oral disc is species specific (McDiarmid and Altig, 1999). All these species use their keratinized oral structures to free organic materials from substrates when they feed. Thus, variation in LTRF configuration likely influences tadpoles’ grazing ability. Tadpoles of *D. minutus* have one ventral and two lateral marginal papillae rows and a LTRF=0/1 (Fig. 4). The oral disc of this species is small (in comparison to the following species) and terminal positioned (i.e. at the tip of the snout). Tadpoles of *T. typhonius* present two marginal papillae rows, LTRF=4(1,2,4)/6(1,6) and the oral disc anteroventrally positioned (Fig. 4). Tadpoles of *S. fuscovarius* have one marginal papillae row, LTRF=2(2)/3(1), and an anteroventrally positioned oral disc (Fig. 4). The oral morphology of bottom-dwelling tadpoles (*L. fuscus* and *P. cuvieri*) is similar to *S. fuscovarius* in LTRF and the number of marginal papillae (Fig. 4). Similar to *S. fuscovarius*, tadpoles of *L. fuscus* have their oral disc anteroventrally positioned. However the oral disc of *P. cuvieri* tadpoles is more ventrally positioned and its third posterior tooth row is one third smaller than the other posterior tooth rows (Rossa-Feres and Nomura, 2006).

**Fig. 5.**
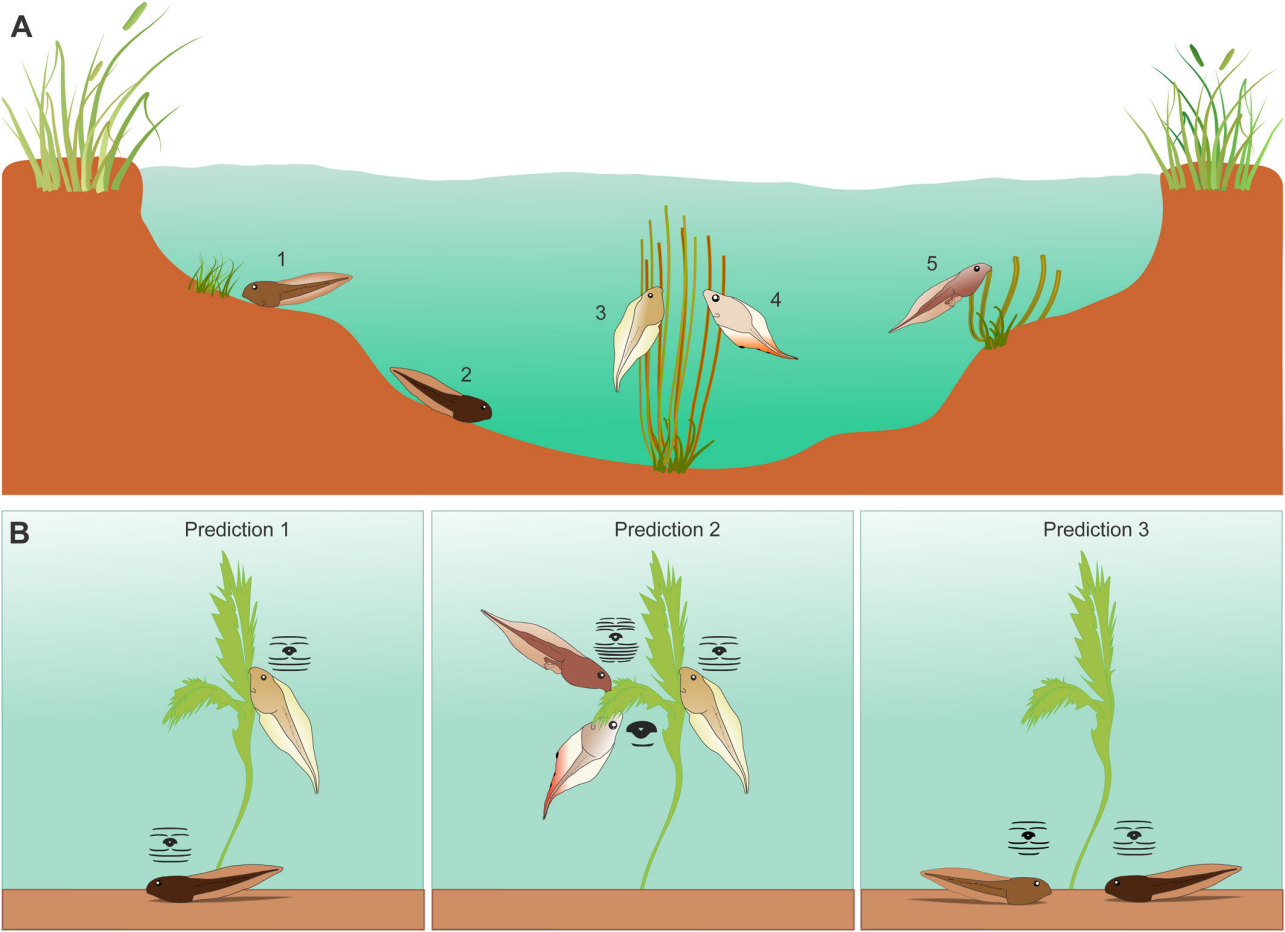
**Schematic drawing of the tadpoles' microhabitats.** (A) The illustration (not drawn to scale) indicates where the tadpoles of the studied species are usually found in natural ponds (both temporary and permanent). Tadpoles close to the bottom are *P. cuvieri* (1), which can also be found close to aquatic vegetation (Eterovick and Sazima, 2000) and *L. fuscus* (2). Tadpoles in the water column are *S. fuscovarius* (3), *D. minutus* (4) and *T. typhonius* (5). Tadpoles of the three species are generally found close to vegetation. Tadpoles of *T. typhonius* are also found in shallow water (Schulze et al., 2015). (B) Schematic for our predictions. From the left to the right, we expect (1) differences in feeding efficiency between species that inhabit different depths of the water column, with higher growth rates on horizontal surfaces for bottom-dwelling tadpoles, and on vertical surfaces for midwater tadpoles. We also expected (2) different foraging abilities depending on the angle where tadpoles forage, among species that inhabit the same microhabitat, but differ substantially in oral morphology. Next we expected (3) similar ability to remove food from substrates for species that inhabit the same microhabitat and have similar oral morphology, with higher growth rates when grazing on horizontal surfaces. Our predictions (B) were based on the microhabitats where the tadpoles occur naturally (A).

The microhabitats where tadpoles of these species most frequently occur also differ (Fig. 5A). The larvae of all these species live in lentic environments, but *L. fuscus* and *P. cuvieri* are bottom-dwelling and occur in shallow microhabitats such as puddles or the margins of ponds (Eterovick and Sazima, 2000; Queiroz et al., 2015; Schulze et al., 2015). Tadpoles of *L. fuscus* are usually observed grazing on organic materials that fell into the water or scraping stems and leaves of aquatic plants (Schulze et al., 2015). Tadpoles of *P. cuvieri* can also be found close to macrophytic aquatic vegetation (Eterovick and Sazima, 2000). Tadpoles of *S. fuscovarius*, *D. minutus* and *T. typhonius* usually occur in the midwater of deeper water bodies, commonly close to vegetation (Vasconcelos and Rossa-Feres, 2005; Vasconcelos et al., 2011; Schulze et al., 2015). Tadpoles of *T. typhonius* can also be found in shallow water (Schulze et al., 2015). Importantly, the tadpoles of these five species can be temporarily and spatially sympatric (Vasconcelos and Rossa-Feres, 2005; Rossa-Feres and Nomura, 2006; dos Santos et al., 2007; Vasconcelos et al., 2011).

### Sampling and experiment design

**Fig. 6.**
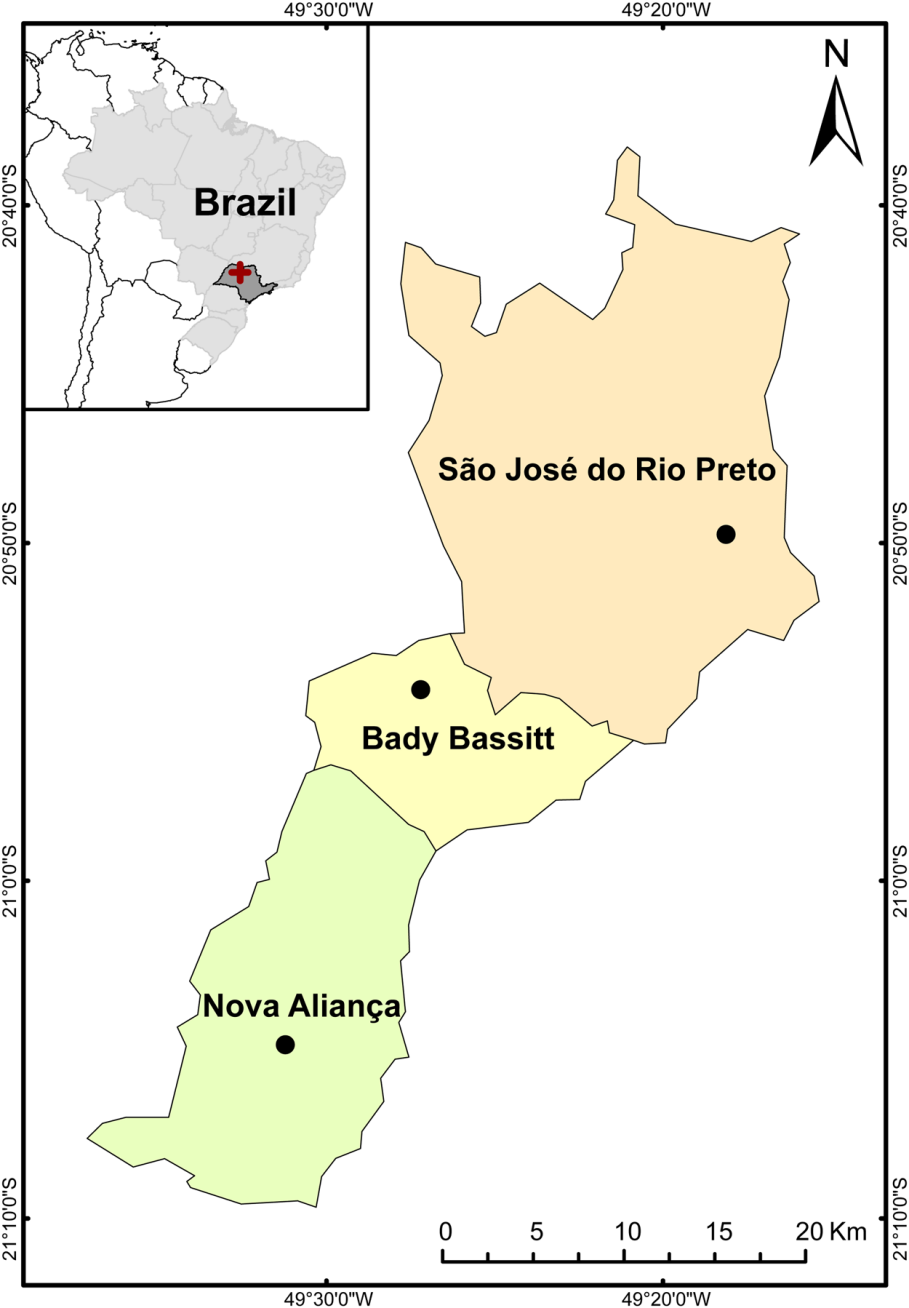
**Sampling area.** The black dots represent the ponds where tadpoles were collected in each municipality (orange, yellow and green areas) of São Paulo state (highlighted in dark gray on the smaller map of Brazil). The cross on the São Paulo state map represents the northwest region where these municipalities are located. Credit: Alba Navarro Lozano.

All tadpoles were collected from ponds in the northwest region of São Paulo state, Brazil (Fig. 6) between October 2015 and February 2016. Tadpoles were acclimatized in the laboratory in polyethylene aquaria for three days before the beginning of the experiments. During the acclimation period, we maintained tadpoles in dechlorinated water, at a 12:12 light:dark photoperiod, with air temperature between 27°C and 28°C and at a water temperature of approximately 25.5°C. Tadpoles also received a powdered commercial algal-based food that contains *Spirulina* and sea algal meal (Sera Micron^®^) *ad libitum*.

Experiments were conducted under the same conditions of luminosity and temperature that the tadpoles experienced during acclimation. However, during the experiment, tadpoles were individually housed in glass aquaria (15×10×13 cm) with the containers' sides covered with a blue adhesive to prohibit visual contact between tadpoles in neighboring aquaria, and to reduce stress that other colors may cause (based on fishes; Maia and Volpato, 2013). The aquaria were filled with dechlorinated water that was gently aerated.

We used tadpoles in similar developmental stage (26–29; Gosner, 1960) during the experiments because tadpoles in this period grow in size (trunk and tail), but do not have well developed limbs. Also during this period there is little differentiation of other anatomical structures, such as oral structures (McDiarmid and Altig, 1999). Within species, we selected tadpoles of similar overall total length.

**Fig. 7.**
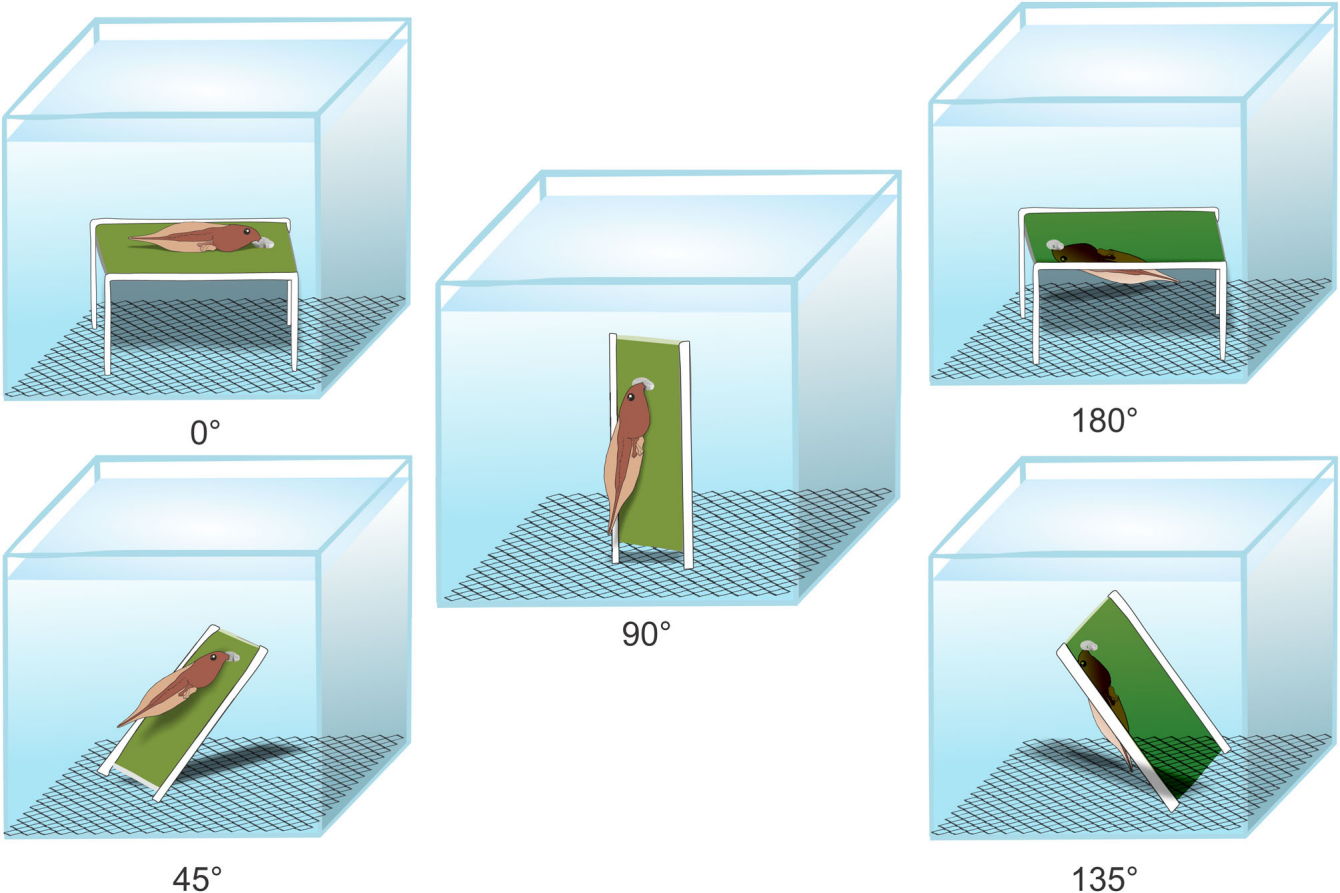
**Experiment design.** Schematic drawing (not to scale) for tadpole feeding during the experiments. Each treatment is represented by one tadpole in a glass aquarium. The green bar represents the food on a microscope slide, oriented by the plastic supports (in white) on a metal screen at the bottom of the aquarium. The spot in the green bar represents the mark that tadpoles leave on the slide after removing food.

To standardize food availability, we followed the protocol used by Venesky et al. (2013). We diluted Sera Micron^®^ in water at a concentration of 40 mg/ml^−1^; then brushed the suspension on one side of a standard glass microscope slide (surface area 19.8 cm²). We repeated the brushing procedure three times for each slide, then allowed the slides to dry for 24 h. We fit the slides in plastic supports, which were then placed on a metal screen at the bottom of each aquaria – this allowed us to orient the slides with food at different angles. Specifically, we placed one slide with food at one of the following five treatment angles per aquarium: 0°, 45°, 90°, 135° and 180° (Fig. 7). We individually tested from 10 to 12 tadpoles of each species at each of the five angles (Table S1). The tadpoles remained in the aquaria for seven days during testing. The experiments were conducted using two species at a time which resulted in 110 different aquaria during a trial (55 aquaria for each species). During the experiments, the test aquaria were placed side by side in four lines and the order of treatments was randomized. We changed the microscope slides containing food twice per day in each test aquarium to assure food was readily available.

We used an analytical balance with readability of 0.1 mg to measure the tadpoles' mass before (hereafter, initial measurement) and after (hereafter, final measurement) the experiments. At the time they were weighed, we photographed each tadpole to estimate its length with ImageJ^®^ software (https://imagej.nih.gov/ij/). We used body length rather than total length to assess tadpole growth because tails, especially fins and flagella, could be easily injured. By taking measurements from photographs instead of directly from the tadpoles, we reduced the amount of time we handled tadpoles and kept them out of the water.

The initial and final measurements of tadpoles were used to calculate the tadpoles' relative growth (*RG*) through Eqn1:(1)
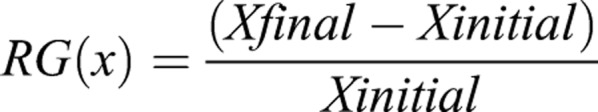


where *X* represents either the mass or body length measurement (Table S1).

We estimated food consumption with pictures taken from each slide after tadpoles fed on them. Using GIMP^®^ software (https://www.gimp.org/), we overlaid the picture with a grid (2×2 mm²) and counted the number of squares filled and not filled with food to calculate the percentage of food consumption (consumption = unfilled squares/total squares).

Tadpoles gain mass directly by consuming food, but also experience energetic costs associated with searching for and removing food from the substrate. As such, we consider feeding efficiency as the growth rate (RG) of individuals, rather than food consumed, to account for differences in energetic costs of removing food from different substrate angles.

### Ethics statement

We collected the tadpoles with approval from the Institute of Environment and Natural Renewable Resources (IBAMA) and Chico Mendes Institute for Biodiversity Conservation (ICMBio) – Authorization and Information System on Biodiversity (SISBio) permit number: 18163-1 to D.C.R.-F. Maintenance of tadpoles and the experiments were in accordance with the Ethics Commission on the Use of Animals (CEUA - 121/2015).

After the experiment, tadpoles were immersed in an anesthetic solution of 2% lidocaine. The tadpoles were then placed in a preservative solution made up of 70% ethanol and 15% formalin. This procedure was important to confirm species identification and also to make the tadpole specimens available for future studies. Preserved specimens were deposited in the Scientific Collection of Universidade Federal de Goiás (UFG).

### Statistical analyses

We first tested whether species with similar oral configuration, but different microhabitat use, differed in efficiency when removing food from substrates at different orientations (Prediction 1, Fig. 5B). For this prediction, we used a two-way ANCOVA to test how relative body mass (response variable) was affected by treatment angle (predictor variable, five levels: 0°, 45°, 90°, 135° and 180°) and by species identity (predictor variable, two levels: *S. fuscovarius –* midwater and *L. fuscus –* bottom-dwelling), using food consumption as the covariate. We also used a two-way ANOVA to test how relative body size (as the response variable) was affected by the same treatments (predictor variable, five levels – angle of substrates) and the same species (predictor variable, two levels) as factors.

We next tested whether species that usually occur at the same depth within the water column, but differ in oral morphology, vary in efficiency when grazing upon substrates at different orientations (Prediction 2, Fig. 5B). We used the same statistical model as above to test this prediction: two-way ANCOVA with relative body mass as the response variable, with angles (five levels: 0°, 45°, 90°, 135°and 180°) and species identity (three levels: *D. minutus*, *S. fuscovarius*, *T. typhonius*) as predictor variables and food consumption as the covariate. When relative size was the response variable, we used a two-way ANOVA with angles (five levels, as above) and species (three levels, as above) as predictor variables.

Finally, we tested whether species that commonly occur in the same depth within the water column and present similar oral morphologies have similar efficiencies feeding at the same substrate orientations (Prediction 3, Fig. 5B). We used the same statistical model to test this prediction: two-way ANCOVA with relative body mass as the response variable, with angles (five levels: 0°, 45°, 90°, 135°and 180°) and species identity (two levels: *P. cuvieri*, *L. fuscus*) as predictor variables and food consumption as the covariate. When relative size was the response variable, we used a two-way ANOVA with angles (five levels, as above) and species (two levels, as above) as predictor variables.

We considered the amount of food consumed as a covariate for relative mass only, once we confirmed that mass was a more direct correlate with food consumed than linear measurements of size (Leips and Travis, 1994; Álvarez and Nicieza, 2002).

When the analyses of variance and covariance identified significant effects of factors on the response variable, we used the post hoc Fischer's test (Least Significant Difference, LSD) to identify specific differences among groups. It was necessary to log transform the relative mass data of species tested in the first and second predictions in order to meet the assumptions of the analyses. We performed all analyses with R software (version 1.0.143, R Development Core Team, 2017).

## Supplementary Material

Supplementary information
