# Supplementary material for: Influence of substrate orientation on tadpoles' feeding efficiency
Source: Biol Open. 2018 Dec 21;8(1):bio037598. doi: 10.1242/bio.037598 (PMC6361219; doi:10.1242/bio.037598)
Supplement: Supplementary information [file biolopen-8-037598-s1.pdf]

Table S1. Mean ± standard deviation (s.d.) of measurements, relative and absolute growth of species.

			Measurements				Relative (%)		Absolute	
			Body size (mm)		Weight (mg)		Body size	Weight	Body size (mm)	Weight (mg)
**Species**	**Treatment**	**n**	**Initial**	**Final**	**Initial**	**Final**	**Mean ± s.d.**	**Mean ± s.d**	**Mean ± s.d**	**Mean ± s.d**
	0°	11	12.03 ± 0.63	15.31 ± 1.15	286.76 ± 40.51	644.89 ± 88.85	0.27 ± 0.10	1.26 ± 0.28	3.30 ± 1.20	358.10 ± 74.30
	45°	9	12.24 ± 1.03	15.06 ± 1.23	276.61 ± 52.05	601.58 ± 111.27	0.23 ± 0.10	1.20 ± 0.42	2.80 ± 1.20	324.90 ± 93.50
T. typhonius	90°	11	12.30 ± 1.12	14.16 ± 0.97	284.89 ± 63.06	565.10 ± 110.62	0.15 ± 0.07	1.00 ± 0.26	1.90 ± 0.80	280.20 ± 70.60
	135°	11	11.84 ± 1.03	14.38 ± 0.77	268.82 ± 54.32	534.76 ± 87.19	0.21 ± 0.07	1.02 ± 0.32	2.50 ± 0.70	265.90 ± 76.20
	180°	11	11.99 ± 0.57	14.68 ± 1.29	268.99 ± 43.23	564.85 ± 130.58	0.22 ± 0.09	1.09 ± 0.38	2.70 ± 1.10	295.90 ± 106.40
			**12.08 ± 0.88**	**14.72 ± 1.08**	**277.21 ± 50.63**	**582.24 ± 105.70**	**0.22 ± 0.09**	**1.11 ± 0.33**	**2.60 ± 1.00**	**305.00 ± 84.20**
	0°	11	10.77 ± 0.96	11.80 ± 0.69	64.65 ± 11.73	356.03 ± 45.14	0.10 ± 0.08	4.75 ± 1.64	1.00 ± 0.70	291.30 ± 51.20
	45°	10	10.09 ± 0.77	11.22 ± 1.08	89.39 ± 14.81	268.90 ± 68.43	0.11 ± 0.05	2.03 ± 0.62	1.10 ± 0.60	179.50 ± 62.10
D. minutus	90°	11	10.31 ± 1.11	11.35 ± 1.22	70.88 ± 17.08	287.67 ± 61.30	0.10 ± 0.07	3.22 ± 1.21	1.00 ± 0.70	216.80 ± 57.80
	135°	11	11.05 ± 0.82	12.06 ± 0.99	73.90 ± 14.50	320.15 ± 64.49	0.09 ± 0.08	3.48 ± 1.33	1.00 ± 0.80	246.20 ± 65.50
	180°	11	10.16 ± 0.96	11.37 ± 0.94	61.68 ± 17.17	293.06 ± 66.84	0.12 ± 0.06	4.02 ± 1.68	1.20 ± 0.60	231.40 ± 65.40
			**10.48 ± 0.92**	**11.56 ± 0.98**	**72.10 ± 15.06**	**305.16 ± 61.24**	**0.10 ± 0.07**	**3.50 ± 1.30**	**1.00 ± 0.70**	**233.00 ± 60.40**
	0°	9	7.71 ± 0.61	9.93 ± 0.80	124.83 ± 37.93	241.63 ± 41.12	0.29 ± 0.08	1.00 ± 0.37	2.20 ± 0.60	116.80 ± 28.70
	45°	10	8.56 ± 0.51	10.35 ± 0.90	162.94 ± 28.57	308.00 ± 79.81	0.20 ± 0.07	0.88 ± 0.32	1.80 ± 0.70	145.00 ± 61.10
S. fuscovarius	90°	8	8.73 ± 0.63	10.20 ± 0.99	149.39 ± 38.57	270.63 ± 86.79	0.16 ± 0.07	0.79 ± 0.21	1.40 ± 0.70	121.20 ± 51.40
	135°	9	8.07 ± 0.52	9.53 ± 1.05	139.03 ± 20.32	228.04 ± 58.05	0.18 ± 0.11	0.64 ± 0.32	1.40 ± 0.90	89.00 ± 48.80
	180°	10	7.56 ± 0.53	9.53 ± 0.82	115.71 ± 11.99	224.02 ± 45.92	0.26 ± 0.12	0.93 ± 0.30	1.90 ± 0.90	108.30 ± 39.90
			**8.13 ± 0.56**	**9.91 ± 0.91**	**138.38 ± 27.48**	**254.46 ± 62.34**	**0.22 ± 0.09**	**0.85 ± 0.30**	**1.70 ± 0.70**	**116.00 ± 45.90**
	0°	9	9.37 ± 0.53	11.41 ± 0.63	134.11 ± 19.22	293.41 ± 61.36	0.21 ± 0.33	1.18 ± 0.07	2.00 ± 0.60	159.30 ± 50.10
	45°	11	9.62 ± 0.43	11.32 ± 0.73	152.75 ± 21.84	298.90 ± 33.57	0.17 ± 0.19	0.97 ± 0.05	1.70 ± 0.50	146.10 ± 22.70
L. fuscus	90°	9	9.55 ± 0.81	11.36 ± 0.58	144.48 ± 34.53	291.24 ± 61.02	0.19 ± 0.57	1.08 ± 0.08	1.80 ± 0.70	146.80 ± 53.30

Biology Open • Supplementary information

	135°	10	9.35 ± 0.57	11.32 ± 0.58	145.15 ± 27.38	285.54 ± 45.98	0.21 ± 0.16	0.98 ± 0.06	1.90 ± 0.60	140.40 ± 25.50
	180°	9	9.58 ± 0.48	11.92 ± 0.52	143.18 ± 15.00	324.16 ± 40.90	0.24 ± 0.35	1.28 ± 0.07	2.30 ± 0.60	180.90 ± 40.40
			**9.49 ± 0.56**	**11.47 ± 0.61**	**143.93 ± 23.59**	**298.65 ± 48.57**	**0.20 ± 0.32**	**1.10 ± 0.07**	**1.90 ± 0.60**	**154.90 ± 38.40**
	0°	12	7.93 ± 0.73	8.62 ± 0.77	103.93 ± 22.91	156.53 ± 32.22	0.09 ± 0.06	0.53 ± 0.29	0.70 ± 0.40	52.60 ± 21.50
	45°	11	7.62 ± 0.49	8.27 ± 0.39	100.55 ± 19.95	148.36 ± 22.33	0.08 ± 0.04	0.49 ± 0.17	0.60 ± 0.30	47.80 ± 14.90
P. cuvieri	90°	12	7.86 ± 0.76	8.66 ± 0.71	105.94 ± 21.32	165.11 ± 28.49	0.10 ± 0.06	0.58 ± 0.19	0.80 ± 0.60	59.10 ± 14.70
	135°	11	7.42 ± 0.65	7.96 ± 0.35	92.94 ± 19.17	123.56 ± 14.08	0.07 ± 0.05	0.36 ± 0.18	0.50 ± 0.30	30.60 ± 11.50
	180°	12	7.38 ± 0.45	7.93 ± 0.51	90.87 ± 17.64	120.23 ± 26.68	0.07 ± 0.06	0.32 ± 0.16	0.50 ± 0.50	29.30 ± 15.60
			**7.64 ± 0.62**	**8.29 ± 0.55**	**98.84 ± 20.20**	**142.76 ± 24.76**	**0.08 ± 0.05**	**0.46 ± 0.20**	**0.60 ± 0.40**	**43.80 ± 15.60**

Measures of weight and body size were taken on the first day of the experiment before releasing the tadpoles into the aquaria (Initial), and on the seventh day, at the end of the experiment (Final). Relative growth was calculated as Eqn1. Absolute growth was calculated as the difference between the initial and final measures of tadpoles. Values in bold represent mean and s.d. for the species, considering all orientations. n: number of tadpoles by treatment.

Biology Open • Supplementary information
